# *Listeria monocytogenes* – How This Pathogen Survives in Food-Production Environments?

**DOI:** 10.3389/fmicb.2022.866462

**Published:** 2022-04-26

**Authors:** Jacek Osek, Beata Lachtara, Kinga Wieczorek

**Affiliations:** Department of Hygiene of Food of Animal Origin, National Veterinary Research Institute, Puławy, Poland

**Keywords:** *Listeria monocytogenes*, stress response, food processing, bacterial persistence, molecular mechanisms, food safety

## Abstract

The foodborne pathogen *Listeria monocytogenes* is the causative agent of human listeriosis, a severe disease, especially dangerous for the elderly, pregnant women, and newborns. Although this infection is comparatively rare, it is often associated with a significant mortality rate of 20–30% worldwide. Therefore, this microorganism has an important impact on food safety. *L. monocytogenes* can adapt, survive and even grow over a wide range of food production environmental stress conditions such as temperatures, low and high pH, high salt concentration, ultraviolet lights, presence of biocides and heavy metals. Furthermore, this bacterium is also able to form biofilm structures on a variety of surfaces in food production environments which makes it difficult to remove and allows it to persist for a long time. This increases the risk of contamination of food production facilities and finally foods. The present review focuses on the key issues related to the molecular mechanisms of the pathogen survival and adaptation to adverse environmental conditions. Knowledge and understanding of the *L. monocytogenes* adaptation approaches to environmental stress factors will have a significant influence on the development of new, efficient, and cost-effective methods of the pathogen control in the food industry, which is critical to ensure food production safety.

## Introduction

*Listeria monocytogenes* is a Gram-positive facultatively anaerobic microorganism, motile at the temperature range of 22–28°C but non-motile above 30°C, characterized by the growth at temperature range from −0.4°C to 45°C, with an optimum temperature of 37°C (Allerberger, 2003). It is able to survive at a relatively low water activity (aW < 0.90) and a broad pH range between 4.6 and 9.5 as well as to tolerate salt conditions up to 20% (Buchanan et al., 1989; Bucur et al., 2018). These growth conditions made these bacteria able to survive and multiply in adverse environmental conditions which are often present at food production facilities (Gray et al., 2006; Ranasinghe et al., 2021). *L. monocytogenes* is therefore an important foodborne pathogen responsible the disease called listeriosis, which can appear as sporadic infections or disease outbreaks with significant mortality rate of 20–30% worldwide (Buchanan et al., 2017). Human infection can occur in three forms, depending on the age of the infected person, its immune status, the amount of ingested bacterial cells, and the virulence properties of the strain: severe or mild invasive listeriosis and non-invasive febrile gastroenteritis (Buchanan et al., 2017). Depending on the severity of the illness, symptoms may last from days to several weeks. Mild symptoms may include a fever, muscle aches, nausea, vomiting, and diarrhea. If the more severe form of listeriosis develops, symptoms may include headache, stiff neck, confusion, loss of balance, and convulsions (Buchanan et al., 2017). The disease is especially dangerous for the elderly, pregnant women, unborn babies and immunocompromised people (de Noordhout et al., 2014). According to the recent European Food Safety Authority (EFSA) and European Center for Disease Prevention and Control (ECDC) common report for year 2020, a total of 1,876 confirmed cases of invasive listeriosis in humans were noted in the European Union member states, with the notification rate of 0.42 cases per 100,000 population and 97.1% hospitalizations (EFSA and ECDC, 2021). In the United States, the Centers for Disease Control and Prevention (CDC) estimate that each year about 1,600 persons are infected with *L. monocytogenes*, with the hospitalization rate of ca. 94% (Scallan et al., 2011).

## *Listeria Monocytogenes* in Food and Food Production Environments

*Listeria monocytogenes* is a ubiquitous bacterium and has been isolated from soil, water, and feed (Dhama et al., 2015). It has been demonstrated that the bacteria can survive in the environment for at least 8 weeks (Watkins and Sleath, 1981; Rodríguez-Campos et al., 2019). Several investigations have shown that *L. monocytogenes* is widely distributed in food processing environments where it is able to persist for a long time due to ineffective cleaning and sanitation (Carpentier and Cerf, 2011; Ferreira et al., 2014; Buchanan et al., 2017). Many strains survive in different food processing conditions which are often characterized with a low humidity or oxygen content, and thus becoming a main source of post-processing contamination (Hoelzer et al., 2012; Ferreira et al., 2014; Malley et al., 2015; Bucur et al., 2018). Persistence of such strains may be contributed by several external factors as poor hygiene practice or ineffective sanitizers but also by the presence of the specific genes in some *L. monocytogenes* strains that are responsible for biofilm production (Nilsson et al., 2011; Harter et al., 2017; Lee et al., 2019; Rodríguez-Campos et al., 2019).

## Adverse Environmental Conditions and Survival of *L. Monocytogenes*

### Low Temperatures

As above-mentioned, *L. monocytogenes* has the ability to grow in a broad range of temperatures (from −0.4°C to 45°C) but also under freezing conditions no significant changes in the live bacteria population was observed (Walker et al., 1990; Gougouli et al., 2008; Nowak et al., 2015). Tolerance to low temperatures resulted in the frequent detection of these bacteria in food products stored under refrigeration conditions (Tasara and Stephan, 2006). The mechanisms of this phenomenon are complex and involve a decrease in the metabolism of the bacterial cells, changes in cell membrane composition, expression of cold shock proteins, and uptake of cryoprotective compounds from the environment (Phadtare et al., 1999; Neunlist et al., 2005; Bucur et al., 2018).

The proper physical condition of the bacterial cell membrane lipids is essential to optimal structural and functional integrity of these membranes (Suutari and Laakso, 1994; Sohlenkamp and Geiger, 2016). Low temperatures affecting the cell lead to reduced membrane lipid fluidity. In response to this stress factor *L. monocytogenes* changes the membrane lipid composition toward an increase in the concentration of unsaturated fatty acids, which prevents formation of a gel-like state that may result in leakage of cytoplasmic content. It also creates the optimal membrane fluidity for enzyme activity and transport across the membrane (Suutari and Laakso, 1994; Gandhi and Chikindas, 2007; NicAogáin and O’Byrne, 2016; Bucur et al., 2018; Santos et al., 2019). Furthermore, the rate of intracellular enzyme activity decreases to the necessary minimum, and the cell fitness is improved (Mastronicolis et al., 2006; NicAogáin and O’Byrne, 2016). For all these purposes the bacteria modulate their genes expression, especially those involved in cell membrane function and synthesis of lipids, carbohydrates and amino acids as well as those involved in biogenesis and motility (Chan et al., 2007b; Cordero et al., 2016).

During exposure to the cold temperature stress conditions, *L. monocytogenes* responds in different ways. The cells increase the accumulation of glycine betaine and carnitine from the environment by a chill-activated transport system (Angelidis and Smith, 2003). Both these organic osmolytes are found in high amounts in various foods which may help to promote the survival and growth of *L. monocytogenes* at lower temperatures (Zeisel et al., 2003). The glycine betaine transporter (Gbu) is an ATP-binding cassette (ABC) transporter that is encoded by the *gbu* operon (Ko and Smith, 1999), whereas transport of carnitine in response to cold shock is depended on the OpuC ABC transporter, the product of the *opuC* operon (Fraser et al., 2000). It has been described that both glycine betaine and carnitine were accumulated much faster by *L. monocytogenes* at 7°C than at 30°C, and their levels increased several times within the cells when grown at 8°C compared to 37°C (Ko et al., 1994; Chan et al., 2007b; Singh et al., 2011).

The role of sigma factor protein σ*^B^* (SigB) in adaptation to cold stress in *L. monocytogenes* has been studied (Chan et al., 2007a; Bucur et al., 2018). The protein is stimulated in response to temperature downshift and the *sigB*-deleted mutant was unable to accumulate solutes such as betaine and carnitine (Chan et al., 2007a; O’Byrne and Karatzas, 2008). It has also been shown that the sigma factor contributed to adaptation in a growth phase-dependent manner, since the absence of SigB protein impaired adaptation of stationary-phase cells to grow at the lower temperature and it was also necessary for efficient accumulation of betaine and carnitine by *L. monocytogenes* as cryoprotectants (Becker et al., 2000). Furthermore, it has been also demonstrated that only some cold-induced genes were under *sigB* control (e.g., *opuCA* gene encoding OpuCA protein with the ATPase-coupled transmembrane transporter activity), whereas other genes responsible for cold shock may be only partially *sigB* factor-dependent (Chan et al., 2007a; Miladi et al., 2017). On the other hand, Utratna et al. (2014) showed that σ*^B^* does not play a key role in survival of *L. monocytogenes* under low temperature stress conditions. These and other authors also demonstrated that *sigB* is activated at 4°C in a manner that was independent of the levels of RsbV and RsbW proteins encoded by the respective *sigB* operon genes (Zhang et al., 2013; Utratna et al., 2014). Interestingly, it has been shown that at 4°C there is a significant correlation between the *prfA* virulence gene regulon responsible for the expression of listeriolysin regulatory protein PrfA and the σ*^B^* regulon, which moderate the activity of PrfA during the host infection (Ollinger et al., 2009; Heras de las et al., 2011).

Other mechanisms of adaptation of *L. monocytogenes* to low temperatures have also been described. One such mechanism is the expression of cold shock-domain family proteins (Csps), which are produced mainly at the temperature range from 4°C to 10°C (Bayles et al., 1996; Phadtare et al., 1999; Hébraud and Guzzo, 2000). Csps are structurally related small proteins (65–70 amino acids long), widely distributed among prokaryotes, with a highly conserved structure, that bind to nucleic acids and regulate the expression of various genes including those involved in stress resistance and virulence, cellular aggregation, and motility in *L. monocytogenes* (Eshwar et al., 2017). Csps stabilize the nucleic acid conformation and act as molecular chaperone that facilitates replication, transcription, and translation at low temperatures (Bucur et al., 2018). Among many Csp proteins identified, CspA contributes to resistance of *L. monocytogenes* to low temperatures (Schmid et al., 2009; Muchaamba et al., 2021).

Another protein belonging to the cold shock-domain family is a low molecular weight (ca. 18 kDa) ferritin-like protein (Flp), which was detected in much higher levels in *L. monocytogenes* cultures kept at −20°C and grown at 4°C compared to the bacteria cultured at 37°C (Hébraud and Guzzo, 2000; Miladi et al., 2012). It has been suggested that regulation of Flp synthesis may occur at the transcriptional level since the increase of *flp* mRNA was detected upon heat and cold shock (Hébraud and Guzzo, 2000).

Pöntinen et al. (2015) described the two-component-system histidine kinases that have been involved in growth of *L. monocytogenes* at low temperatures and play a role in adaptation to cold stress. Two genes, *yycGF* and *lisRK*, responsible for the bacterial adaptation to cold stress conditions, were identified (Pöntinen et al., 2015). The authors suggested that YycF protein encoded by the *yycF* gene was more involved in the early stage of cells survival, whereas the *lisRK* product was rather responsible for a longer cold acclimation (Pöntinen et al., 2015).

### High Temperatures

Thermal treatment is one of the methods that have been applied in food production and preservation to prevent or limit the growth of pathogenic microorganisms, including *L. monocytogenes* (Bucur et al., 2018; Ricci et al., 2021). However, the efficacy of high temperature used for inactivation of *Listeria* during food processing may be limited due to their natural resistance to elevated thermal conditions above 45°C (Arioli et al., 2019). It has been described that the total heat inactivation of *L. monocytogenes* requires the temperature range from 55°C for 10 min to 65°C for 12 s, respectively (Smelt and Brul, 2014). There are several external factors that have an influence on resistance of *L. monocytogenes* to heat such as bacterial cells’ age, previous growth and stress conditions, composition of food, strain serotype, etc. (Sörqvist, 1994; Doyle et al., 2001). Cells in the stationary growth phase are generally more resistant to thermal stress than those in log-phase (Doyle et al., 2001). It has been shown that some components present in foods or growth media protect the bacterial cells from heat damage either by stimulation of cellular membrane production or expression of stress-related proteins (Casadei et al., 1998; Juneja et al., 1998). Shen et al. (2014) demonstrated that *L. monocytogenes* isolates of serotype 1/2a showed a relatively low resistance to elevated temperatures, whereas strains classified to serotypes 1/2b and 4b were more heat-resistant although differences among strains of the same serotypes were also noted.

In response to elevated temperature the bacterial cells show increased production of heat shock proteins (HSPs) (Bucur et al., 2018; Wiktorczyk-Kapischke et al., 2021). In *L. monocytogenes* three classes of heat shock-associated genes have been identified (Van der Veen et al., 2007; Wiktorczyk-Kapischke et al., 2021). Several genes (*grpE*, *dnaK*, *dnaJ*, *groEL*, and *groES*) encode the class I HSPs that act as intracellular chaperones and their expression increases when heat-induced denatured proteins accumulate in the bacterial cytoplasm (Bucur et al., 2018). The class I HSP genes are controlled by the HrcA repressor which negatively regulates expression of this kind of stress response genes (Nair et al., 2000; Hu et al., 2007; Roncarati and Scarlato, 2017). The main role of this class of heat shock proteins is to stabilize and repair partially denatured proteins and to prevent their intracellular aggregation under heat stress conditions (Hendrick and Hartl, 1993; Hartl and Hayer-Hartl, 2002). The class II HSP genes encode general stress proteins whose transcription is dependent on the alternative sigma factor SigB in different growth-inhibiting conditions (Hecker et al., 1996; Nair et al., 2000). Class III heat shock genes, including *clpP*, *clpE*, and *clpC* operons, are negatively regulated by the class III HSP gene regulator CtsR which contains domains that are highly conserved among low GC Gram-positive bacteria, including *L. monocytogenes* (Karatzas et al., 2003). The ClpC and ClpE proteins possess ATPase activity and are classified to the heat shock protein Clp family of highly conserved molecular chaperones, whereas the serine protease ClpP is a protein possessing proteolytic properties (Schirmer et al., 1996). At increased temperature, McsB kinase, the product of the *mcsB* gene of the *clpC* operon, modifies CtsR conformation preventing its binding with gene promoters. As a result, RNA-s32 polymerase binds with promoters leading to gene expression, and CtsR is degraded (Wiktorczyk-Kapischke et al., 2021). It has been shown that ClpC expression is negatively controlled at the transcription level by the cAMP protein receptor PrfA (Heras de las et al., 2011).

### Low pH

A low pH environment may be present in food that has undergone acidification, one of the methods of food preservation widely applied to dairy products, meat and vegetables, and is primarily the results of fermentation by bacteria either present in the raw food or added as starter cultures (Hill et al., 2017). Furthermore, *L. monocytogenes* meets acid conditions in the gastrointestinal tract of the host (NicAogáin and O’Byrne, 2016). The bacteria are able to survive in a low pH of the environment which is generated by artificially induced acidification during acid sanitation (Cotter and Hill, 2003). Low pH increases the concentration of hydrogen protons, which results in the inhibition of microbial growth (Ryan et al., 2008). Furthermore, it has been shown that low pH not only allows the bacteria to survive but also increases its virulence and provides *L. monocytogenes* higher protection against other adverse environmental conditions (Rodríguez-López et al., 2018).

The bacteria are able to adapt to this low pH environment by means of different mechanisms. Pre-exposure of *L. monocytogenes* to mild acidic pH of 5.5 for 2 h induces the acid tolerance response (ATR), the process in which the bacteria showed increased resistance to lethal acidic, temperature (52°C), salinity (25–30% NaCl) and alcoholic (15%) shocks (Phan-Thanh et al., 2000). These effects were even more evident when the bacteria adapted to acid gradually (Koutsoumanis et al., 2003). O’Driscoll et al. (1996) also demonstrated that the bacteria exhibited a significant adaptive acid tolerance response following a 1-h exposure to pH 5.5, which is capable of protecting cells from severe acid stress (pH 3.5). It has been also suggested that low pH conditions may have the influence on the selection of *L. monocytogenes* mutants with increased virulence properties (O’Driscoll et al., 1996).

*Listeria monocytogenes* uses a variety of metabolic and homeostatic mechanisms to maintain the intracellular pH within a range that is optimal for its growth and survival (Arcari et al., 2020). It is able to increase cytoplasmic buffer capacity through the glutamate decarboxylase (GAD) system or with the action of an internal proton pump (Cotter et al., 2001; Ryan et al., 2008). The GAD mechanism is considered as one of the major mechanisms responsible for the maintenance of the intracellular homeostasis (Cotter et al., 2001). GAD in most *L. monocytogenes* strains is encoded by five genes, of which three genes (*gadD1*, *gadD2*, and *gadD3*) encode decarboxylases, whereas two other genes (*gadT1* and *gadT2*) are responsible for production of antiporters (Melo et al., 2015). All these five genes are organized in three separate genetic loci: *gadD1T1*, *gadT2D2*, and *gadD3* (Cotter et al., 2005). The glutamate decarboxylase enzyme promotes the irreversible conversion of cytosolic glutamate to a neutral compound, the γ-aminobutyrate (GABA) (Cotter and Hill, 2003). During the GABA synthesis the intracellular proton level decreases resulted with the subsequent alkalization of the environment and increase of the pH inside of the *L. monocytogenes* cell. Furthermore, the extracellular GABA excretion leads to the slight neutralization of the pH outside the cell due to the exchange of extracellular glutamate for the more alkaline GABA, and finally the restart of the metabolic pathway (Cotter and Hill, 2003).

Another cell system that protect Gram positive bacteria from low pH is based on the arginine deiminase (ADI) pathway (Soares and Knuckley, 2016). ADI, with the participation of two other enzymes, carbamoyltransferase and carbamate kinase, all encoded by the *arcABC* operon, converts external arginine to ornithine which is then extracellularly transported in an energy-independent manner by a membrane-bound antiporter encoded by the *arcD* gene (Ryan et al., 2009). The level of ammonia produced as a byproduct of the system combines with intracellular protons to yield NH4^+^ maintaining the intracellular level of the cytoplasmic pH, thereby protecting the *L. monocytogenes* cell from adverse acidic extracellular environments (Cotter and Hill, 2003; Ryan et al., 2009; Matereke and Okoh, 2020). It has been shown that the transcription of *arcA* and *argR* genes is both sigma factor protein σ*^B^* (SigB) and protein receptor PrfA-dependent (Ryan et al., 2009).

In addition to GAD and ADI systems, other proton pumps such as F_0_F_1_-ATPase have also been suggested as active mechanisms to maintain *L. monocytogenes* homeostasis in low (mild) pH environments (Cotter et al., 2000). ATP produced during arginine conversion under ADI mechanisms is used by F_0_F_1_-ATPase to generate a proton gradient, enabling H^+^ expulsion and homeostasis restoration (Smith et al., 2013). The enzyme consists of two distinct domains: the membrane domain F_0_, which is a channel for proton translocation, and the cytoplasmic domain F_1_, responsible for catalyzing ATP synthesis and hydrolysis (Smith et al., 2013).

The above-described low pH adaptation systems, i.e., ATR, GAD, and proton extrusion (F_1_F_0_-ATPase), act at the same time and ensure survival and adaptation to acid stress conditions of *L. monocytogenes* (Wiktorczyk-Kapischke et al., 2021).

*Listeria monocytogenes* also possesses two-component signal transduction system that plays a role in response to environmental stress conditions, including low pH (Cotter and Hill, 2003). This system typically contains two genes, *lisR* and *lisK*, encoding cytoplasmic response regulator and a membrane-associated histidine kinase sensor, respectively. The LisRK signal transduction system is able to sense pH changes in the environment by histidine kinase, and the response regulator enables the cell to respond by altering the relevant gene expression (Cotter et al., 1999). It has been shown that a LisRK transposon mutant of *L. monocytogenes* was more sensitive to low pH than the wild type strain during the logarithmic phase of growth but more acid resistant during stationary phase (Cotter et al., 1999).

### High pH

In food production environment there are several alkaline stress factors, which are sublethal for *L. monocytogenes* and are mainly associated with the use of detergents and disinfectants (Beales, 2004). This bacterium has developed many strategies to withstand adverse high pH-related conditions and as a result they become more cross-resistant to subsequent other, usually more severe, stress factors, such as thermal, alkali, and ethanol stresses or cleaning procedures (Taormina and Beuchat, 2001). Alkali adaptation mechanisms of *L. monocytogenes* may be significant in the persistence of these pathogenic bacteria in food industry equipment and premises in the presence of alkaline-based detergents used in food processing environments (Giotis et al., 2007; Shen et al., 2016).

Alkali conditions, which may be present in the environment due to the use of detergents and disinfectants, can induce the solubilization of bacterial surface proteins, resulting in exposure of hydrophobic sites of lipids to the extracellular factors (Jacobsohn et al., 1992; Giotis et al., 2009). Such conditions may also directly change the structure of the cell membrane by saponification of membrane lipids or alteration of the membrane fatty acids ratio (Giotis et al., 2007). These changes induce damages that significantly disrupt cell metabolism and structure, preventing effective interactions between bacterial cells and their environment (Almakhlafi et al., 1995).

Generally, to resist alkali damage and maintain cytoplasmic pH at optimal values, *L. monocytogenes* responds in different ways. One of them is increased metabolic production of intracellular acids through deamination of amino acids and fermentation of sugars (Padan et al., 2005; Giotis et al., 2010). The bacteria are also able to induce transporters and enzymes directly responsible for proton retention and cell surface modifications (Soni et al., 2011). It has been proved that monovalent cation-proton antiporters are essential to maintain a neutral cytoplasmic pH and, therefore, to allow the bacterial growth under alkaline conditions (Gardan et al., 2003a; Giotis et al., 2010). In addition, the acidic cell wall polymers such as teichuronic acid and teichuronopeptides contribute to pH homeostasis, and provide a passive barrier to ion flux and elevation of the cytoplasmic buffering capacity (Krulwich et al., 1997; Gardan et al., 2003a).

A scanning electron microscopy study of *L. monocytogenes* exposed to sublethal alkaline stress performed by Giotis et al. (2009) showed that the bacterial cells significantly changed their length, radius and volume that may be associated with increased survival of *Listeria* in such adverse environments. Furthermore, in alkaline conditions, *L. monocytogenes* cells develop higher proportions of branched-chain fatty acids, including more anteiso forms that are important in adaptation to high pH (Giotis et al., 2007).

### Osmotic Shock

*Listeria monocytogenes* can survive in elevated osmolarity and has the ability to grow even in media supplemented with 12% NaCl and can tolerate adverse salt conditions up to 20% (Bucur et al., 2018). High NaCl concentrations suppress bacterial growth by decreasing water activity in surrounding environment, enhancing plasmolysis and consequently resulting in decreased intracellular turgor pressure and finally, inhibiting the bacterial amplification (Amezaga et al., 1995). In addition to increasing osmotic pressure, NaCl decreases electrochemical potential across the cell membrane, thus, disturbing ATP production by oxidative phosphorylation (Shabala et al., 2006). The response of *L. monocytogenes* to osmotic stress is called osmoadaptation, a biphasic process consisting of primary and secondary response mechanisms (Hill et al., 2002). The primary phase of adaptation of the bacteria to elevated osmolarity covers physiological changes of the cells, which maintain their turgor by increasing the uptake of potassium ions (K^+^) and its counterion, glutamate, into the cell, and then replacing part of the accumulated K^+^ with low-molecular-weight molecules known as compatible solutes or osmolytes in the second stage of osmoadaptation (Sleator et al., 2003). *L. monocytogenes* possesses two K^+^ transporters, which play a main role in adaptation to high salt concentration: a high affinity KdpABC transporter system, and a low affinity system encoded by the *lmo0993* gene (Kallipolitis and Ingmer, 2001; Brøndsted et al., 2003; Ballal et al., 2007).

Uptake of compatible solutes by *L. monocytogenes* as a response to elevated osmolarity helps the bacteria to restore turgor pressure and cell volume and stabilize cell protein structure and functions (Kallipolitis and Ingmer, 2001; Brøndsted et al., 2003; Sleator et al., 2003). Several compatible solutes which promote both salt and low temperature tolerances in *L. monocytogenes* have been identified, including betaine, carnitine, proline, proline betaine, acetylcarnitine, gamma-butyrobetaine, and 3-dimethylsulfoniopropionate (Bayles and Wilkinson, 2000). Among them, betaine has the strongest effect on reduced growth under high osmotic conditions (Beumer et al., 1994). The presence of the osmolytes resulted in an up to 2.6-fold increase in growth rate of salt-stressed *L. monocytogenes* cells compared to stressed cells without any osmoprotectants (Bayles and Wilkinson, 2000).

It has been described that the main carnitine transport system is encoded by the *opuCABCD* operon, the glycine betaine by *gbuABC*, whereas the glycine betaine uptake system depends on the *betL* gene (Chan et al., 2007b). The expression of genes encoding betaine, carnitine and proline transporters are transcriptionally regulated by general stress sigma factor σ*^B^* (Sleator et al., 2003; Bae et al., 2012). In more detail, the product of the *opuCABCD* operon (OpuCA) releases energy during ATP hydrolysis which is needed for transport of the substrate by a complex consisting of the two transmembrane proteins OpuCB and OpuCD as well as a solute binding protein OpuCC (Fraser et al., 2000). The products of the remaining genes (*betL* and *gbuABC*) are involved in the primary response to the elevated level of NaCl and toleration of *L. monocytogenes* to a long-term osmolarity, respectively (Sleator et al., 2003). The strains possessing mutations in these genes showed reduced growth under high osmotic condition (Okada et al., 2008). It has been suggested that compatible solutes may play a dual role in osmoregulation process of the bacterial cells: firstly, they are involved in restoring of cell volume and, secondly, they stabilize protein structure and function under these adverse environmental conditions (Cayley et al., 1992). It has also been shown that the general stress protein Ctc of *L. monocytogenes* is involved in osmotolerance in the absence of any compatible solutes in the environment (Gardan et al., 2003b).

Several studies indicated that most osmotolerance-associated genes present in *L. monocytogenes* have also been activated during other stress environmental conditions such as low temperature and low pH generated during artificial food acidification as well as play a role in virulence in a mouse model (Okada et al., 2008). On the other hand, in response to osmotic stress, *L. monocytogenes* expresses genes other than those associated with osmolyte accumulation, such as *csp* encoding cold shock-domain family proteins (Csps) (Schmid et al., 2009). These proteins, mainly CspA and CspD, have chaperon activity and facilitate the repair of DNA lesions made by high concentrations of NaCl (Dmitrieva et al., 2004). Apart from stress survival functions, Csp proteins are also involved in cell regulatory networks, playing a crucial role in the regulation of virulence functions of *L. monocytogenes*, especially those connected with invasion and listeriolysin (LLO) secretion (Loepfe et al., 2010; Schärer et al., 2013).

Bae et al. (2012) described that the presence of osmotic conditions in *L. monocytogenes* environment decreased expression of genes associated with phosphoenolpyruvate (PEP)-dependent phosphotransferase carbohydrate systems (PTS), including those related to uptake of β-glucoside, galactitol, fructose, and cellobiose. This has an influence on a significantly lower growth rate of the bacteria and reduced uptake of carbohydrates under osmotic stress conditions (Stoll and Goebel, 2010).

### High Hydrostatic Pressure

High hydrostatic pressure (HPP) is a food preservation technology used as an alternative to thermal processing. It is widely applied in the meat industry for microbial inactivation of both food spoilage microorganisms and foodborne pathogens, and it is conducted at room temperature, which enhances the safety and shelf life of food (Huang et al., 2014). The pressures applied for sterilization depends on the kind of food and potentially present microorganisms and usually ranges between 250 and 700 MPa (mainly 400 and 600 MPa) for a few seconds to 10 min (Bucur et al., 2018). Primary effects of HPP on bacterial cells are an increase in the permeability of the cell membrane, the disruption of the protein structure and function, and finally, inhibition of the physiological activities of the treated microorganisms (Huang et al., 2014).

It has been reported that HPP causes morphological, structural, physiological, and genetic changes or damages to *L. monocytogenes* cells (Ferreira et al., 2016). However, several factors influence the resistance of these bacteria to high hydrostatic pressure. Cells in the stationary phase of growth are much more resistant to pressures above 200 MPa than those in the exponential phase (Huang et al., 2014). Furthermore, resistance of *L. monocytogenes* to HPP depends on the strain and the type, composition and matrix of food products (Bruschi et al., 2017). It has also been shown that higher salt concentrations in food may induce uptake of compatible solutes, which in turn stabilizes cells during HPP (Abe, 2007). It has been shown that pressure-induced damage of the cell membrane has an influence on the Mg^2+^ leakage from the cell and therefore destabilization of ribosome structure, whereas Ca^2+^ strengthens the outer membrane of bacterial cells and make the bacteria more resistant to HPP (Niven et al., 1999; Gänzle and Liu, 2015). However, it has been reported that *L. monocytogenes* treated with HPP up to 550 MPa, resulting in still viable cells, was able to recover and grow during the storage under refrigeration conditions (Bozoglu et al., 2004; Valdramidis et al., 2015).

Several genes and mechanisms that may play a role in recovery from HPP damage of *L. monocytogenes* have been identified. High pressure processing induces expression of genes associated with DNA repair, transcription and translation protein complexes, cell division, general protein secretion system, flagella assemblage and motility, chemotaxis, and membrane and cell wall biosynthesis pathways (Bowman et al., 2008). On the other hand, HPP suppresses a wide range of energy production and conversion, carbohydrate metabolism and virulence-associated genes (Bucur et al., 2018). An important aspect of the stress-induced survival is induction of the general stress response mediated by the above-mentioned sigma factor protein σ*^B^* (SigB) which can activate several protective genes under stressful conditions (Wemekamp-Kamphuis et al., 2004; Guerreiro et al., 2020). However, it has also been suggested that HPP may reduce expression of the sigma factor SigB and part of the *sigB* regulon (Bucur et al., 2018).

It has been shown that HPP also affected genes controlled by the transcription factor CodY, a known global regulator of metabolic genes, including the *prfA* gene regulon responsible for the expression of listeriolysin regulatory protein PrfA (Lobel et al., 2015). Another gene induced by HPP is *cspL* encoding a cold-shock protein, which supports the earlier observations that HPP also induces cross-resistance to other stress factors and conditions like heat, acid, and oxidative stress (Karatzas and Bennik, 2002; Bucur et al., 2018). Furthermore, mutations in CtsR, a class III stress genes repressor, have also been connected to spontaneous resistance of *L. monocytogenes* to HPP (Karatzas et al., 2003). Such mutants, characterized by a stable resistance to HPP, showed point mutations, insertions or deletions in the *ctsR* gene that down-regulated its activity. This feature was connected with increased expression of the *clpB*, *clpC*, *clpE*, and *clpP* genes, encoding a large protein complex Clp (caseinolytic protein) with both proteolytic and chaperone activities (Karatzas et al., 2003; Van Boeijen et al., 2010). Clp proteases are able to degradate damaged or denatured proteins forming in the bacterial cells during HPP treatment that are potentially harmful for *L. monocytogenes*; thus, they increase tolerance of the bacteria to high pressure (Tomoyasu et al., 2001). However, there is also evidence that isolates which did not have such *ctsR* and other genetic changes still showed resistance to HPP treatment (Karatzas et al., 2005; Chen et al., 2009).

It has been shown that after HPP treatment *L. monocytogenes* up-regulates the major PEP-PTS, especially fructose-, mannose-, galactitol-, cellobiose-, and ascorbate-specific, involved in the sugars transport (Stoll and Goebel, 2010; Duru et al., 2021). Furthermore, the cell-division-related genes (*divIC*, *dicIVA*, *ftsE*, and *ftsX*) were also down-regulated, whereas the peptidoglycan-synthesis genes responsible for cell-wall repair (*murG*, *murC*, and *pbp2A*) were upregulated (Duru et al., 2021). Thus, it seems that the bacterial tolerance response to HPP is complex and needs further investigations.

### Ultraviolet Light

Pulsed ultraviolet light (PUV) is a non-thermal approach that has a high potential for decontamination of food, water, and air in food production environments (Gómez-López et al., 2007). For this purpose, ultra-short duration pulses of an intense broadband emission spectrum that is rich in UV-C light (200–280 nm band) of the highest germicidal efficacy is used. This UV spectrum mediates bacterial inactivation through several mechanisms, including damage to the bacterial cells in the form of pyrimidine dimers and the loss of cytoplasmic contents post-light absorption (Gómez-López et al., 2007; Rastogi et al., 2010). The main bactericidal effect of UV-C light is caused by DNA damage as a consequence of the formation of photoproducts such as cyclobutane-pyrimidine dimers (CPDs), pyrimidine 6-4 pyrimidone photoproducts (6-4PPs), and their Dewar isomers (Rastogi et al., 2010). Furthermore, UV light also has photophysical and photothermal effects on bacterial cells due to the absorption of the high energy light pulses resulting in leakage of cellular content (Gómez-López et al., 2007). However, during UV treatment, CDPs are the most common lesions in the bacterial genome, which have the principal effect on the cell development and survival, especially that connected with transcription, DNA replication and cell cycle progression (Beauchamp and Lacroix, 2012).

The efficacy of UV treatment in decontamination of food surfaces depends on many factors, such as the kind of food, distance of the product to the light source, energy level given by number and frequency of the light pulses, time applied for the UV treatment, level of contamination, and others (Gómez-López et al., 2007). It has been shown that *L. monocytogenes* is more resistant to UV-C light than other bacterial pathogens, such as *E. coli* (Beauchamp and Lacroix, 2012). The presence of organic materials such as food debris on stainless steel surfaces and NaCl content in ultraviolet treated foods have an influence on the efficacy of UV-C radiation on *L. monocytogenes* due to low ability of this light to penetrate organic substances (Bernbom et al., 2011). Other studies have also shown that the presence of salt in brine increases the amount of UV-C necessary for inactivation of *L. monocytogenes* in fluid (McKinney et al., 2009b). Similar increased resistance to ultraviolet light was observed for the bacterial cells grown *in vitro* in a low pH, presence of antibiotics, or disinfectants (McKinney et al., 2009a; Naitali et al., 2009).

There is very little information related to detailed molecular mechanisms of UV resistance in *L. monocytogenes*. It seems that UV-C resistance is not general stress sigma factor protein σ*^B^*-dependent (Gayán et al., 2015). Another study of Uesugi et al. (2016) revealed that overall changes in gene expression resulting from ultraviolet treatment of *L. monocytogenes* 10403S strain were low. However, a number of genes encoding stress proteins, motility and transcriptional regulators were up-regulated by the UV exposure, although no induction of the *lmo0588* gene, responsible for deoxyribodipyridimine photolyse activity induced by light was observed (Uesugi et al., 2016). Photolyase, the product of the *lmo0588* gene, plays an important role in photoreactivation, i.e., the recovery of bacteria sublethally injured by UV light due to subsequent exposure of visible light (Gómez-López et al., 2007). It has been shown that, during photoreactivation, photolyase binds and repairs the pyrimidine DNA lesions using light energy absorbed by its chromophores (Sinha and Häder, 2002).

It has also been described that, among the putative stress response genes located on plasmids of *L. monocytogenes*, the *uvrX* gene being a part of the Y-family DNA polymerase, plays a role in response of the cells exposed to UV stress (Naditz et al., 2019; Cortes et al., 2020). A similar finding was described for the chromosomally encoded gene *uvrA*, which was shown to be necessary for the bacterial UV stress survival (Kim et al., 2006). Recently, Anast and Schmitz-Esser (2021) identified several *L. monocytogenes* plasmids that confer increased UV stress tolerance, although their precise role in this phenomenon needs further investigations.

### Heavy Metals

Certain heavy metals such as copper, zinc, and iron in trace amounts are essential for bacterial survival and play a role of co-factors for a broad range of *L. monocytogenes* cellular proteins but the same metals at higher concentrations often become toxic (Jesse et al., 2014; Parsons et al., 2019). Other heavy metals (e.g., arsenic and cadmium) are probably not necessary for cellular functions and seem to be toxic in any concentration (Jesse et al., 2014; Parsons et al., 2019). However, *L. monocytogenes* possesses various mechanisms to maintain its cellular heavy metal homeostasis, avoid poisoning, and thus, survive in diverse environmental niches (McLaughlin et al., 2011; Jesse et al., 2014). In this section, resistance to the main toxic heavy metals, i.e., cadmium and arsenic, is discussed.

#### Cadmium Resistance

It has been reported that approximately 50% or more of tested *L. monocytogenes* isolates from foods and food processing plants were resistant to cadmium (Ratani et al., 2012). On the other hand, there is also information that most strains were susceptible to this metal at concentration of 64 μg/ml cadmium sulfate (Margolles et al., 2001). Resistance to cadmium is encoded by different genetic determinants often located on mobile genetic elements (mainly plasmids) (Parsons et al., 2019). At least five molecular mechanisms have been identified that contribute to cadmium resistance, although resistant strains lacking these genetic determinants have also been identified suggesting that there are yet unidentified means of metal tolerance (Lee et al., 2013). The first cadmium molecular resistance gene described in *L. monocytogenes* was the *cadA1* located on plasmid-borne Tn5422 transposon, which is responsible for the activity of efflux P-type ATPase pumps (Lebrun et al., 1994). Few strains harbor Tn*5422*-associated *cadA1* chromosomally (Hingston et al., 2019). A second putative cadmium resistance sequence, *cadA2*, was initially detected on the large plasmid pLI100 of *L. innocua* and then discovered on the approximately 80 kb plasmid pLM80 of *L. monocytogenes* H7858, a strain implicated in a large listeriosis outbreak in the United States in 1998–1999 (Nelson et al., 2004). It was also found that pLM80-associated *cadAC* (*cadA2*) is part of a putative composite transposon that also harbors genes for resistance to benzalkonium chloride (Elhanafi et al., 2010). In addition, pLM80 determines resistance to toxic triphenylmethane dyes such as crystal violet and malachite green *via* the *tmr* gene (Dutta et al., 2014). The third cadmium resistance gene, *cadA3*, is carried on the integrative and conjugative element (ICE) of *L. monocytogenes* EGD-e (Elhanafi et al., 2010). Finally, the *cadA4* and *cadA5* are on the large chromosomally located *Listeria* Genomic Island 2 (LGI2) and LGI2-1 islands, respectively (Parsons et al., 2020). The *cadA3, cadA4*, and *cadA5* genes have so far been identified only on chromosome (Kuenne et al., 2010; Lee et al., 2017; Parsons et al., 2019). It has also been found that LGI2 harbors arsenic resistance cassette comprising of *arsD1A1R1D2R2A2B1B2* genes (Lee et al., 2013). Comparison of the *cadA1-cadA4* sequences revealed that the first three conferred a high level of resistance to cadmium (MIC > 140 μg/ml), whereas *cadA4* was responsible for relatively lower resistance levels (MIC < 70 μg/ml) (Lee et al., 2013). Interestingly, LGI2 genetic element has been mainly connected with *L. monocytogenes* classified into hypervirulent clones of clonal complexes CC1 and CC2 as well as CC4 (Lee et al., 2017). Furthermore, strains with multiple *cadA* variant determinants have been identified (Lee et al., 2013). On the other hand, several *L. monocytogenes* isolates lacking any of the four cadmium resistance determinants were detected, which suggests the presence of one or more unidentified cadmium resistance genes (Ratani et al., 2012; Lee et al., 2013).

It has been described that the prevalence of the known cadmium resistance molecular determinants was serotype-related: *cadA1* was more common in *L. monocytogenes* isolates of serotypes 1/2a and 1/2b than 4b from food and food-processing environment, while *cadA2* was mainly associated with strains of serotype 4b (Ratani et al., 2012; Lachtara et al., 2021). However, several cadmium-resistant isolates lacking the known *cadA* determinants were classified to serotype 4b, which suggests that such strains may possess other than *cadA*-encoded resistance mechanisms (Ratani et al., 2012; Chmielowska et al., 2021). Furthermore, another study showed that cadmium resistance was more common among persistent *L. monocytogenes* strains, i.e., those repeatedly isolated from foods than among those recovered sporadically (Harvey and Gilmour, 2001).

#### Arsenic Resistance

One of the most important mechanisms responsible for arsenic resistance in *L. monocytogenes* appears to be encoded by genes carried on the above-mentioned LGI2, primarily associated with strains of serotype 4b, especially of the hypervirulent clones CC1, CC2, and CC4 (Lee et al., 2017). Molecular analysis of arsenic-resistant isolates harboring LGI2 revealed that this island was inserted in at least eight different locations, primarily within open reading frames (Lee et al., 2017). Strains classified to serotypes 1/2a, 1/2b, and 1/2c rarely carried the LGI2 insert, thus, are usually arsenic-sensitive (Hingston et al., 2019). It has been shown that the arsenic resistance cassettes contain three (*arsRBC*) to five (*arsRDABC*) genes (Rosen, 1999; Kuenne et al., 2010). The products of the *arsA* (encoding ATPase) and *arsB* (responsible for a membrane transporter) genes are an ATP-dependent anion pump that exports arsenite out of the bacterial cells (Tisa and Rosen, 1990). Furthermore, the *arsA* gene product is also able to act independently as a passive transporter of arsenite (Rosen, 1999). The *arsC* gene encodes a reductase that is responsible for the conversion of arsenate to arsenite, which is then extruded by ArsA or the ArsA/ArsB complex (Tisa and Rosen, 1990). The *arsA*, *arsB*, and *arsC* genes are regulated by two regulatory proteins encoded by *arsR* and *arsD* (Wu and Rosen, 1993). Interestingly, the LGI2 insert present in *L. monocytogenes* harbors several additional genes, including the putative cadmium resistance determinant *cadA4* mentioned above (Parsons et al., 2019).

### Biocides

#### Resistance to Quaternary Ammonium Compounds

Increased tolerance of *L. monocytogenes* to disinfectants (biocides) has been deeply studied in relation to quaternary ammonium compounds (QACs), such as benzalkonium chloride (BC), widely applied in food production and health care environments as well as in households due to their effectiveness, low toxicity, and non-corrosive properties (Gerba, 2015; Kode et al., 2021). QACs are usually used in concentrations ranging from 200 ppm to 400 ppm on food-contact surfaces; however, some formulations also have 1,000 ppm concentrations (Aryal and Muriana, 2019). The antimicrobial effectiveness of QACs and other biocides may be affected by the surface structure of the food production equipment to which bacteria are attached, an uneven distribution of disinfectants on the decontaminated surfaces, too high dilution of disinfectants due to presence of water on the equipment surfaces, or the presence of organic pollutants resulting from insufficient cleaning before disinfection (Duze et al., 2021). Biofilms, often produced by *L. monocytogenes*, also have an impact on resistance of these bacteria against QAC disinfectants (Duze et al., 2021). Most QACs are aerobically biodegradable and their concentrations in the environment varied resulting in the formation of concentration gradients (Tezel and Pavlostathis, 2015). This may lead to the generation of selective pressure for adaptation or acquisition of resistance genes of initially susceptible *L. monocytogenes* strains (Tezel and Pavlostathis, 2015; Conficoni et al., 2016).

Quaternary ammonium compounds are active agents that interact with the cytoplasmic membrane of bacteria, including *L. monocytogenes*, and also able to interact with intracellular targets as well as to bind to DNA (Zinchenko et al., 2004). It has been shown that at low concentrations (0.5 to 5 mg/L) they are characterized by bacteriostatic properties whereas, at concentrations of 10–50 mg/L, QACs are bacteriocidal for the same bacteria, depending upon the formulation (Gerba, 2015). However, there are studies showing that several *L. monocytogenes* isolates were resistant to BC at a concentration of 1,000 mg/L after 24 h of exposure time (Mohamed et al., 2018).

The action of these biocides involves absorption of QACs by the bacterial cell and penetration of the cell wall, reaction with the cytoplasmic membrane and its disruption, leakage of intracellular components, degradation of proteins and DNA, and finally lysis of bacterial cell wall by autolytic enzymes (McDonnell, 2017). It has been shown that the effectiveness of QACs seems to be not different between persistent and non-persistent *L. monocytogenes* strains isolated from food production environments (Ruckerl et al., 2014; Magalhães et al., 2016). However, there are also reports showing that persistent isolates from food processing plants and ecosystems exhibited higher resistance to QACs than non-persistent ones, especially strains classified to serotype 1/2c (Ortiz et al., 2016; Meier et al., 2017). Studies of Haubert et al. (2019) demonstrated that all 50 *L. monocytogenes* isolates from food tested were resistant to benzalkonium chloride, and more than 50% of these tolerant strains displayed cross-resistance to cadmium. Such correlation between BC and cadmium resistances has been previously reported by other authors, especially among *L. monocytogenes* of serotypes 1/2a and 1/2b (Mullapudi et al., 2008; Ratani et al., 2012).

It has been observed that exposure of *L. monocytogenes* to increasing concentrations of BC resulted in adaptation to higher levels of this and other biocides (Yu et al., 2018; Noll et al., 2020). These studies demonstrated that repeated exposure to subinhibitory concentrations of QACs and prolonged environmental persistence of tolerant strains may facilitate the development of bacterial resistance to these biocides over time. Thus, selection of BC-tolerant bacteria increases the ability of *L. monocytogenes* to survive under treatment with higher concentrations of the same biocide (Ortiz et al., 2014; Tezel and Pavlostathis, 2015). Consequently, this process further contributes to the persistence of *L. monocytogenes* in the food processing environments (Møretrø et al., 2017).

The major molecular mechanisms of quaternary ammonium compounds resistance in *L. monocytogenes* involve several efflux pump systems, including the three-gene cassette *bcrABC* associated with BC tolerance (Noll et al., 2020). These cover two endogenous multidrug efflux pump genes (multidrug resistant *Listeria*, *mdrL*, and *Listeria* drug efflux, *lde*) of the major facilitator superfamily and efflux pump genes (*bcrABC* cassette, *qacH*, *emrE*, and *emrC*) located on mobile genetic elements (Duze et al., 2021). It has been shown that the two major efflux pump genes, *mdrL* and *lde*, have been identified in almost all *L. monocytogenes* serotypes, and enhanced expression of these two endogenous efflux pumps, especially MdrL, resulted in BC resistance (Martínez-Suárez et al., 2016; Yu et al., 2018; Haubert et al., 2019; Jiang et al., 2019). However, the main role of these gene products is detoxification of macrolides, cefotaxime, heavy metals, and ethidium bromide (*mdrL*), and fluoroquinolones, ethidium bromide, and acridine orange (*lde*), respectively (Mata et al., 2000).

Among the four efflux pump genes located on mobile genetic elements, the *bcrABC* cassette was firstly identified in *L. monocytogenes* responsible for the multistate listeriosis outbreaks in 1998–1999 in the United States (Elhanafi et al., 2010). In most isolates, this cassette is located on the pLM80 plasmid but has also been identified on chromosome (Dutta et al., 2013). These authors have also demonstrated that, in BAC-tolerant *L. monocytogenes* from various sources, the *bcrABC* cassette was present in 98.6% of isolates (Dutta et al., 2013).

The *qacH* efflux pump gene is located on a chromosomally integrated Tn*6188* transposon of 5,117 bp in size and consists of three transposase genes (*tnpABC*) as well as genes encoding a putative transcriptional regulator and QacH, a small multidrug resistance protein family (SMR) transporter associated with export of BC in bacteria (Müller et al., 2014). The significant expression of *qacH*-encoded efflux pumps has been shown in the presence of benzalkonium chloride and the *qacH* deletion mutants had lower tolerance to BC than wild type strains (Müller et al., 2014). It has also been described that QacH protein confers higher tolerance to other QACs and ethidium bromide (Müller et al., 2014). A study of Meier et al. (2017) demonstrated that the majority of Swiss and Finnish *L. monocytogenes* 1/2c clinical and food isolates resistant to BC were *qacH*-positive, although a subset of BC-resistant strains lacked genes for efflux pumps currently known to confer BC resistance. Similar observations were described by other authors (Ortiz et al., 2014; Ebner et al., 2015; Møretrø et al., 2017).

Other efflux pump genes responsible for the increased tolerance of *L. monocytogenes* to QAC are *emrE* and *emrC* sequences, located on the LGI1 genomic mobile island and pLMST6 plasmid, respectively (Kovacevic et al., 2016; Kremer et al., 2017). The *emrE* gene was first described in a study on *L. monocytogenes* isolates responsible for the deadliest listeriosis outbreak in Canada in 2008 (Kovacevic et al., 2016). During this investigation it was found that strains possessing the LGI1 island with the *emrE* sequence was characterized by a significantly improved bacterial growth in the presence of QACs compared to the adaptation and growth of genetically similar strains but lacking LGI1. Furthermore, the expression of *emrE* and several other genes on the LGI1 genomic island was induced in the presence of BC, whereas deletion of the *emrE* gene resulted in reduced bacterial growth and survival in the presence of QACs (Kovacevic et al., 2016).

The *emrC* QAC resistance gene, carried by plasmid pLMST6, was identified in *L. monocytogenes* of sequence type ST6 strains, isolated from adults suffering from listeriosis with meningitis (Kremer et al., 2017). Interestingly, Kropac et al. (2019) demonstrated that the plasmid pLMST6 was not associated with increased tolerance to benzalkonium chloride, but rather increased tolerance to other types of QAC-based biocides. Furthermore, pLMST6 plasmid had no impact on the sensitivity of *L. monocytogenes* to non-QAC disinfectants or on resistance of isolates to ampicillin, tetracycline and gentamicin (Kropac et al., 2019).

#### Resistance to Other Biocides

Chlorine-based disinfectants such as sodium hypochlorite, chlorine dioxide gas or aqueous chlorine dioxide are used in food industry to control *L. monocytogenes* contamination (Vaid et al., 2010). These chemicals possess fast and strong oxidizing properties and interact with bacterial cell wall membranes, mainly phospholipids, or penetrate directly into the cell wall where they form *N*-chloro groups that react with the bacterial metabolism due to the interference with key enzymes (Wei et al., 1985; Denyer and Stewart, 1988). The efficacy of chlorine-based disinfectants seems to be bacterial cell age-dependent since younger cultures (24 h) are more resistant than older ones (48 h) (El-Kest and Marth, 1988). Furthermore, in *L. monocytogenes* biofilms the efficacy of chlorine solutions depends on the material on which the biofilm is formed, e.g., bacteria are more easily destroyed when grown on stainless steel surfaces compared to those grown on polyvinyl chloride or Teflon surfaces (Bremer et al., 2002; Pan et al., 2006).

The effect of chlorine-based disinfectants against *L. monocytogenes* also depends on the chemical compounds used. It has been shown that chlorine dioxide is less toxic, more effective at low concentrations and needs a shorter reaction time than chlorine alone (Chang et al., 2000). One of the main disadvantages of chlorine-based biocides is the formation of toxic disinfection-by-products, especially when they are dissolved in water containing organic matter which is often the case in food production environments (Vaid et al., 2010; Duze et al., 2021). Such products, including trihalomethanes and haloacetic acids, are potential carcinogens and have been associated with various health problems (Chang et al., 2000; Rand et al., 2007).

Acid compounds, like chlorine-based disinfectants, are strong oxidizers and have an effective antibacterial properties (Chang et al., 2000; Vaid et al., 2010). They interfere with cellular phospholipids and cytosolic intracellular material causing irreversible damage (e.g., disruption of proton motive force) and subsequent cells death (Denyer and Stewart, 1988).

## Biofilms

*Listeria monocytogenes* is able to attach to a variety of surfaces in food production environments, including stainless steel, polystyrene or glass, and then to form biofilms (Rodríguez-López et al., 2018). This is a serious concern for food safety because biofilm-contaminated food environments may serve as source of pathogenic bacteria for food products and finally for consumers (Colagiorgi et al., 2017). Bacterial cells in biofilms are embedded in a self-produced matrix of extracellular material, composed of extracellular DNA, proteins, polysaccharides, and other inorganic molecules, called extracellular component matrix (ECM) (Colagiorgi et al., 2017). In the *L. monocytogenes* biofilm matrix, various extracellular polymeric substances (EPSs) have been identified, with different polysaccharides (mainly teichoic acid), proteins, and extracellular DNA (Colagiorgi et al., 2016). It has been shown that *L. monocytogenes* biofilms are strongly influenced by temperature, bacterial strain, incubation time, medium, and the nature of the adhesion surface (Borucki et al., 2003; Midelet et al., 2006; Harvey et al., 2007; Tresse et al., 2007; Di Bonaventura et al., 2008; Mazaheri et al., 2021). Di Bonaventura et al. (2008) and Tomičić et al. (2016) observed that *L. monocytogenes* was able to form biofilms at 4 and 12°C with higher levels on glass compared to the more hydrophobic stainless steel and polystyrene. Furthermore, in both cases, the production of biofilms was significantly higher at 37°C than at 4°C. These authors suggested that these results were not due to a different cellular physiology but rather to a reduced growth of bacteria (Di Bonaventura et al., 2008; Tomičić et al., 2016). On the other hand, Bonsaglia et al. (2014) observed biofilm formation at 4°C on different surfaces, with higher levels of biofilm on stainless steel and glass compared to polystyrene. Similar data were presented earlier by Norwood and Gilmour (2001) who showed that some *L. monocytogenes* strains were able to adhere in the same way at 4°C and 30°C. Other studies suggested that cold-adapted *L. monocytogenes*, stored at −20°C for 6 and 24 months, was characterized by increased adhesion and biofilm formation on various abiotic surfaces (Slama et al., 2012).

It has been shown that flagella-mediated motility plays a key role in both initial surface attachment and subsequent biofilm formation by *L. monocytogenes* and the *flaA* mutants displayed reduced colonization ability (Lemon et al., 2007; Todhanakasem and Young, 2008; Gorski et al., 2009; Doghri et al., 2021; Mazaheri et al., 2021). Since temperature regulates flagella expression in *L. monocytogenes*, it is clear that this factor has a strong influence on biofilm formation (Todhanakasem and Young, 2008). It has been demonstrated that this pathogen is flagellated and motile at temperatures < 30°C, and not at all or much less flagellated and motile at temperatures above 30°C (Gründling et al., 2004). However, *L. monocytogenes* is also able to attach to abiotic surfaces through a flagella-independent binding process, which is not related to a temperature (Tresse et al., 2009).

A correlation between *L. monocytogenes* serotypes or clones and biofilm formation has been investigated but no clear dependence was detected (Borucki et al., 2003; Harvey et al., 2007). Doijad et al. (2015) tested the ability of 98 clinical and food isolates classified to serotypes 1/2a, 1/2b, and 4b to form a biofilm. Most of the strains (63.3%) were classified as weak biofilm producers, whereas the remaining isolates were defined as moderate and strong (9.2% of each) biofilm formers (Doijad et al., 2015). Interestingly, none of the strains of 4b serotype exhibited strong biofilm formation. It has been also shown that strong biofilm-forming isolates developed biofilm structures within 24 h on surfaces important in food industries such as stainless steel, ceramic tiles, high-density polyethylene plastics, polyvinyl chloride pipes, and glass (Doijad et al., 2015). Using whole-genome sequencing data from 166 environmental and food-related *L. monocytogenes* biofilm-forming isolates, it has been suggested that serotype-specific differences in biofilm development can be linked to the presence of stress survival islet 1 (SSI-1) (Keeney et al., 2018). In this study, strains of serotype 1/2b, the majority of which contained SSI-1, formed the strongest biofilms, while isolates classified to serotype 4b, which only some of them were SSI-1-positive, were the weakest biofilms producers.

Investigations performed by Takahashi et al. (2009) on 71 *L. monocytogenes* of food origin revealed a significant correlation between isolates of lineage I (serotypes l/2b and 4b) but not strains of lineage II (serotypes 1/2a and l/2c) and biofilm formation. However, it was also found that isolates classified to the same clonal lineage produced different levels of biofilms, which may suggest that environmental factors are involved in this process (Carpentier and Chassaing, 2004). On the other hand, Borucki et al. (2003) found a higher biofilm-forming ability for *L. monocytogenes* isolates of lineage II. A reason for these different results obtained by several authors may be caused by the differences in methods applied and the various strains used for the study.

Significant differences in gene expression between biofilm-forming and planktonic *L. monocytogenes* bacterial cells, especially those involved in expression of internalins (InlA and InlC) and listeriolysin O (LLO), have been observed (Lourenço et al., 2013; Mata et al., 2015; Gilmartin et al., 2016). Isolates with mutations in the *inlA* gene, which resulted in the reduced length of InlA protein, demonstrated enhanced biofilm forming abilities but a lower virulence potential compared to the strains possessing full-length InlA (Franciosa et al., 2009). Such mutations occur more commonly among food isolates than in strains responsible for human infections (Nightingale et al., 2005).

Lemon et al. (2010) found that *prfA*, the transcriptional activator of virulence genes, promotes biofilm formation in *L. monocytogenes*. Although in *prfA* negative mutants the flagellar motility remains intact and the cells are able to attach to abiotic surfaces, they are defective in next stages of biofilm formation (Lemon et al., 2010).

Schwab et al. (2005) investigated the role of the alternative stress sigma factor σ*^B^* in biofilm formation and revealed that initial attachment of both wild type and mutant *L. monocytogenes* to the stainless steel surface was the same, but the number of *sigB*-deficient strain on the surface was significantly lower than the wild type after 48 h or 72 h of incubation.

Recently, Fan et al. (2020) studied a role of the two-component chemotactic system encoded by the *cheA/cheY* genes, located immediately downstream of the *flaA* flagellin gene. The *cheY* knockout mutant showed decreased biofilm formation ability along with reduced cell-surface hydrophobicity compared to wild type strain. Similar results were also obtained by Li et al. (2021) who showed that *cheA* and *cheY* are key genes in the formation of *L. monocytogenes* aggregates *in vitro*.

The *agrBDCA* operon present in *L. monocytogenes* consists of genes that code for AgrD, an auto-inducing peptide, AgrB, a protein involved in processing the peptide, AgrC, a two-component histidine kinase, and AgrA, a response regulator (Miller and Bassler, 2001). It has been shown that *agr* system, that is involved in quorum sensing, has a strong influence on biofilm formation as mutations in *agrA* and *agrD* display reduction in their ability to form biofilms compared to the wild type strains under both static and dynamic conditions (Rieu et al., 2007; Riedel et al., 2009; Zetzmann et al., 2016). Pieta et al. (2014) studied the presence and expression of the *agrA* gene in *L. monocytogenes* of serotypes 1/2a and 4b, grown at 7°C and 37°C. The authors found that the gene was not detected in strains of serotype 4b, and its transcription level in strains of serotype 1/2a was lower at 7°C compared to 37°C.

Alonso et al. (2014) identified 38 genetic loci possibly involved in *L. monocytogenes* biofilm formation when grown at 35°C. Among them, the D-alanylation pathway genes *dltABCD* and the phosphate-sensing two component system *phoPR* were important in this process since the deletion mutants showed decreased ability to produce biofilms. It may suggest that D-alanylation of lipoteichoic acids mediated by the gene products of the *dltABCD* operon and the phosphate-sensing *phoPR* system play a significant role for *L. monocytogenes* to form biofilms.

A role of autoinducer (AI-2) molecules and the *luxS* gene in quorum sensing and biofilm production by *L. monocytogenes* was tested by Garmyn et al. (2009). The authors revealed that S-ribosylhomocysteinase encoded by *luxS* catalyzes the hydrolysis of S-ribosylhomocysteine to homocysteine and 4,5-dihydroxy-2,3-pentadione, precursor molecules of AI-2, and thus are involved in biofilm formation (Bonsaglia et al., 2014). Mutation in *luxS* led to production of a denser biofilm and better attachment by a *luxS*-deficient mutant to a glass surface compared to the wild type strain (Belval et al., 2006; Sela et al., 2006). Furthermore, the culture supernatants of *luxS* mutants were shown to accumulate S-adenosyl homocysteine and S-ribosyl homocysteine, the AI-2 precursor molecules (Belval et al., 2006).

## Resistance and Persistence

Persistent *L. monocytogenes* strains have been defined as isolates (clones) that are repeatedly cultured from the same source or ecological niche over time (Palaiodimou et al., 2021; Unrath et al., 2021). Such isolates have indistinguishable molecular background as tested by genome-based approaches, e.g., pulsed-field gel electrophoresis (PFGE) or recently, next generation sequencing (NGS) (Fox et al., 2011; Brown et al., 2021; Unrath et al., 2021). Persistence of *L. monocytogenes* is due to different characteristics of such isolates, e.g., tolerance to sanitizers, ability to grow at low temperatures, resistance to heavy metals, or ability to develop biofilm (Kathariou, 2002; Gandhi and Chikindas, 2007; Carpentier and Cerf, 2011; Palaiodimou et al., 2021). Persistent isolates present an important challenge to food producers, as they are associated with cross-contamination of food products because they are hardly or not at all eliminated from food production environments (Palaiodimou et al., 2021).

Several genetic determinants have been suggested to play a role in persistence of *L. monocytogenes*; however, the nature of the role of these mechanisms to the persistence phenomenon remains still poorly understood (Palaiodimou et al., 2021). This may be due to difficulties in creating appropriate conditions under *in vitro* studies that accurately reflect the natural environment in food production facilities. Overall, there is a lack of conclusive evidence on whether persistent strains are more resistant to particular stress conditions compared to sporadic strains from similar sources (Taylor and Stasiewicz, 2019).

Persistent *L. monocytogenes* strains have been isolated from food processing environments after cleaning and disinfection (Lundén et al., 2003; Soumet et al., 2005). The relationship between resistance to various biocides and persistence of certain subtypes of *L. monocytogenes* in different food processing environments has been studied but no clear correlation was identified (Heir et al., 2004; Kastbjerg and Gram, 2009; Ferreira et al., 2014). On the other hand, there are investigations that showed that there is a link between resistance to benzalkonium chloride and persistence of some strains, especially those positive for the *bcrABC* gene cassette (Elhanafi et al., 2010; Martínez-Suárez et al., 2016; Ortiz et al., 2016; Cherifi et al., 2018; Cooper et al., 2021).

A correlation between biofilm formation and persistence of *L. monocytogenes* in the food production environments has been investigated by several authors (Lundén et al., 2000; Borucki et al., 2003; Colagiorgi et al., 2017; Rodríguez-López et al., 2018; Lee et al., 2019; Lianou et al., 2020; Fagerlund et al., 2021; Mazaheri et al., 2021; Unrath et al., 2021). Generally, persistent strains have shown increased biofilm formation in relation to non-persistent strains (Borucki et al., 2003). It has been also observed that biofilms produced on stainless steel surfaces by persistent strains are thicker than those formed by strains found only sporadically (Lundén et al., 2000). Norwood and Gilmour (2001) tested the adherence capability to stainless steel surface of two *L. monocytogenes* strains with and without persistent ability. It was shown that mean counts of adherent cells over a 24-h period at 25°C were significantly higher for persistent strains (Norwood and Gilmour, 2001). Similar observations were noted by Lundén et al. (2000) and Borucki et al. (2003). Further studies confirmed that persistent *L. monocytogenes* genotypes were often associated with higher survival and biofilm formation capacity in the presence of sublethal concentrations of benzalkonium chloride (Maury et al., 2019). On the other hand, other authors have shown that there were no clear associations between biofilm formation efficiency and persistent or prevalent genotypes (Djordjevic et al., 2002; Harvey et al., 2007; Jensen et al., 2007; Lee et al., 2019).

In a study of Wen et al. (2011) persistent *L. monocytogenes* of serotype 4b were shown to be extremely resistant to high temperatures and pressure stresses. Resistance to cadmium has been more often noted among persistent clones compared with their sporadically contaminating counterparts (Harvey and Gilmour, 2001; Parsons et al., 2020). On the other hand, Palaiodimou et al. (2021) have shown that high frequencies of known cadmium resistance cassettes were almost equally present among both persistent (86%) and presumed non-persistent (83%) *L. monocytogenes* populations. However, their results suggest that the *cadA1* gene was more common in persisters, whereas the *cadA4* sequence, which provides lower tolerance to cadmium than *cadA1*, was only carried in non-persistent isolates (Palaiodimou et al., 2021).

Two *L. monocytogenes* stress survival islets (SSIs) which provide benefits to growth and/or survival under suboptimal or stress conditions, such as low pH (SSI-1), alkaline pH (SSI-2) or oxidative stress conditions (both islets), are usually overexpressed among persistent populations identified in food production environments (Ryan et al., 2010; Harter et al., 2017; Palaiodimou et al., 2021). Interestingly, it has been suggested that persistent *L. monocytogenes* may possess a lower virulent potential due to the presence of truncated *inlA* gene, frequent lack of additional virulence factors such as LIPI-3 and LIPI-4 and the mutations in the *prfA* gene (Ortiz et al., 2016; Palaiodimou et al., 2021).

Taylor and Stasiewicz (2019) compared the influence of different stress conditions (10% of NaCl; different concentrations of benzalkonium chloride; and energy sources) on the growth of persistent and sporadic *L. monocytogenes* strains of food origin and confirmed observations of other authors that there was not a significant difference in growth rate or ability to grow for isolates of persistent strains compared to sporadic strains for any treatments at 37°C.

## Novel *L. Monocytogenes* Control Strategies

Several strategies for *L. monocytogenes* elimination from food chain have been developed and applied in the food industry (Khan et al., 2016; Rothrock et al., 2019; Zhang et al., 2021). One of them is irradiation processing technology with gamma irradiation. The approach has been shown to be safe and is a proven method used worldwide for food product preservation (Lacroix and Ouattara, 2000; Maherani et al., 2016). Food irradiation involves exposing food to gamma radiation to induce the demise of *L. monocytogenes* and other bacteria that can cause food poisoning or food spoilage (Lacroix and Follett, 2015).

Another method of elimination of the pathogens from food and food production environments is application of ozone, the eco-focused method which is categorized as generally recognized as safe (GRAS) (Panebianco et al., 2021). It has been recently shown application of gaseous ozone at 50 ppm on planktonic cells and biofilm of reference and food-related *L. monocytogenes* strains resulted in over 3 log_10_ CFU/ml reduction of bacterial load after 10 min (Panebianco et al., 2021). Furthermore, a complete inactivation of planktonic cells after 6 h of treatment as well as a significant decrease of the biofilm biomass were observed. Thus, the use of gaseous ozone is a promising method of *L. monocytogenes* contamination control on both food contact surfaces and on the final products (Botta et al., 2020).

One novel alternative biological method of *L. monocytogenes* control along the food chain is the use of phages (Kawacka et al., 2020). Phages are considered an effective tool against bacterial pathogens as they only target their specific organism and do not interfere with other microorganisms. This is especially important in the production of fermented foods as they do not have a negative influence on the sensory properties of the final product (Sadekuzzaman et al., 2017; Moye et al., 2018). There are commercial phage-based products which are successfully applied in food industry to control *L. monocytogenes*. One of them is ListShield™ (Intralytics, Columbia, MD, United States), a cocktail of six different lytic bacteriophages that is specifically designed for treating foods that are high risk for *L. monocytogenes* contamination, like ready-to-eat meat (RTE) products (Perera et al., 2015). It has been shown that ListShield™ significantly reduced by 82–99% the number of *L. monocytogenes* in different kinds of RTE food (Perera et al., 2015). In the case of smoked salmon, the phages completely eliminated the pathogen in both the naturally contaminated and experimentally contaminated samples without affecting the organoleptic quality of the food. ListShield™ can also be used to eliminate or significantly reduce the levels of *L. monocytogenes* on non-food contact equipment, surfaces, etc., in food processing plants and other food establishments (Ishaq et al., 2020).

LISTEXTM P100 (Micreos, Hague, Netherlands) is also approved by the FDA and recommended by EFSA as a phage cocktail product for the reduction of *L. monocytogenes* on meat and poultry foods during processing or in the final product (EFSA, 2016). The effectiveness of the broad host range bacteriophage P100 present in this products was tested for the reduction of *L. monocytogenes* in inoculated samples at different temperatures and the maximum decrease of the number of the pathogen was achieved at the level of 4.44 log CFU/g in contaminated food samples compared with the control group (Miguéis et al., 2017). When LISTEX™ P100 was applied on biofilms formed on stainless steel, 3.5–5.4 log CFU/cm^2^ reductions were observed depending on which of the 21 *L. monocytogenes* strains were tested (Soni and Nannapaneni, 2010; Gray et al., 2018). According to the EFSA opinion, LISTEX™ P100 is completely harmless, effective and does not contribute to antibacterial resistance (EFSA, 2016).

Despite many positive results and recommendations, the routine using of bacteriophages in food production industry and final food products is allowed only in some countries and regulations relate only to individual bacteriophage products (Kawacka et al., 2020). Despite the wide use of LISTEXTM P100 (e.g., in the United States, Canada and Switzerland), its acceptance as a processing aid in multiple countries (including Australia, New Zealand, Israel, Switzerland, Canada even in one EU member, Netherlands) (Aprea et al., 2018; Połaska and Sokołowska, 2019), and the previously stated positive opinion of EFSA, the EU has not approved this product for application in food industry (EFSA, 2016). Thus, further studies on the safety and effectiveness of phage-based preparation against *L. monocytogenes* in foods as well as monitoring of the occurrence of phage-resistant strains in food processing plants are needed.

## Conclusion

*Listeria monocytogenes* is an important foodborne pathogen responsible for severe sporadic infections or disease outbreaks with high case fatality rates worldwide (Scallan et al., 2011; de Noordhout et al., 2014; Buchanan et al., 2017). These ubiquitous bacteria have been isolated from soil, water, feed, and food production environments, where they can survive and persist for a long time. Resistance of such strains to different food processing conditions is contributed to by several external factors such as poor hygiene practice or ineffective sanitizations, but also by the presence of diverse genetic determinants that are responsible for resistance to extreme temperatures, pH, heavy metals, biocides, and the ability to form biofilms. Although there is much knowledge about the mechanisms of stress responses and resistance to adverse conditions of *L. monocytogenes*, these pathogenic bacteria are still present in the food production environments and pose a severe threat to consumers. Thus, knowledge and understanding of the mechanisms of *L. monocytogenes* adaptation to environmental stress factors will have a significant influence on the development of new, efficient, and cost-effective methods of the pathogen control in the food industry which is critical to ensure food production safety.

## Author Contributions

JO, BL, and KW conceptualized the idea of the manuscript. BL and KW collected relevant literature. JO drafted the manuscript. All authors reviewed, edited the manuscript, and read and approved the final version of the manuscript.

## Conflict of Interest

The authors declare that the research was conducted in the absence of any commercial or financial relationships that could be construed as a potential conflict of interest.

## Publisher’s Note

All claims expressed in this article are solely those of the authors and do not necessarily represent those of their affiliated organizations, or those of the publisher, the editors and the reviewers. Any product that may be evaluated in this article, or claim that may be made by its manufacturer, is not guaranteed or endorsed by the publisher.
